# Cumulative Low Back Load at Work as a Risk Factor of Low Back Pain: A Prospective Cohort Study

**DOI:** 10.1007/s10926-012-9375-z

**Published:** 2012-06-21

**Authors:** Pieter Coenen, Idsart Kingma, Cécile R. L. Boot, Jos W. R. Twisk, Paulien M. Bongers, Jaap H. van Dieën

**Affiliations:** 1 MOVE Research Institute Amsterdam, Faculty of Human Movement Sciences, VU University Amsterdam, Van der Boechorststraat 9, 1081 BT Amsterdam, The Netherlands; 2 Body@Work, Research Center on Physical Activity, Work and Health, Amsterdam, The Netherlands; 3 EMGO, Institute for Health and Care Research, Department of Public and Occupational Health, VU University Medical Center, van der Boechorststraat 7, 1081 BT Amsterdam, The Netherlands; 4 EMGO, Institute for Health and Care Research, Department of Clinical Epidemiology and Biostatistics, VU University Medical Centre, PO BOX 7057, 1007 MB Amsterdam, The Netherlands; 5 TNO Healthy Living, Polarisavenue 151, 2132 JJ Hoofddorp, The Netherlands

**Keywords:** Low back loading, Ergonomics, Workers, Longitudinal studies, Observational studies

## Abstract

*Purpose* Much research has been performed on physical exposures during work (e.g. lifting, trunk flexion or body vibrations) as risk factors for low back pain (LBP), however results are inconsistent. Information on the effect of doses (e.g. spinal force or low back moments) on LBP may be more reliable but is lacking yet. The aim of the present study was to investigate the prospective relationship of cumulative low back loads (CLBL) with LBP and to compare the association of this mechanical load measure to exposure measures used previously. *Methods* The current study was part of the Study on Musculoskeletal disorders, Absenteeism and Health (SMASH) study in which 1,745 workers completed questionnaires. Physical load at the workplace was assessed by video-observations and force measurements. These measures were used to calculate CLBL. Furthermore, a 3-year follow-up was conducted to assess the occurrence of LBP. Logistic regressions were performed to assess associations of CLBL and physical risk factors established earlier (i.e. lifting and working in a flexed posture) with LBP. Furthermore, CLBL and the risk factors combined were assessed as predictors in logistic regression analyses to assess the association with LBP. *Results* Results showed that CLBL is a significant risk factor for LBP (OR: 2.06 (1.32–3.20)). Furthermore, CLBL had a more consistent association with LBP than two of the three risk factors reported earlier. *Conclusions* From these results it can be concluded that CLBL is a risk factor for the occurrence of LBP, having a more consistent association with LBP compared to most risk factors reported earlier.

## Introduction

In the past decades, epidemiological studies have contributed to our understanding of the aetiology of low back pain (LBP). Risk factors for the occurrence of LBP, can roughly be divided into: personal factors (e.g. age, smoking habits, physical capacity and body weight [1–4]), psychosocial factors (e.g. stress, social support and job satisfaction [5–8]), and physical factors [9–12]. Of these physical factors, twisting, bending, lifting and whole body vibrations are the most frequently reported ones associated with LBP [13–15]. Nevertheless, some recent reviews suggest that the evidence for a relationship between physical risk factors and LBP is not convincing [2, 11], and generally, data on exposure-response relationships are scarce and incomplete. It can be argued that the relationships of these physical exposures with LBP might be less reliable than the relationship of low back load dose (i.e. the effect that physical exposure has in the human body) with LBP, since different exposures (e.g. lifting and bending) affect the same dose [16]. While parameters of low back load, like low back moments or spine compression forces, could be used as such dose measures, information on the dose–response relationship of LBP is limited. Marras et al. investigated the predictive value of a variety of parameters of low back loading with the risk of LBP [17, 18]. Moreover, some other studies suggest that cumulative loads acting on the spine may contribute to LBP [19–21], however, these results are based on retrospective studies. Dose–response relationships obtained from prospective cohort studies have never been reported. The aim of the present study therefore was to investigate the association of cumulative low back load (CLBL) with LBP, in a large prospective cohort study. Furthermore, the association with LBP of this dose estimate will be compared to associations for exposures reported earlier to be related to LBP. We hypothesized that CLBL, quantified in terms of low back moments, is associated with LBP and that the association of this dose measure with LBP is more consistent than that of exposure measures that were previously established as risk factors for LBP.

## Study Population and Methods

### Population

Data used in this study are part of the Study on Musculoskeletal disorders, Absenteeism and Health (SMASH), a prospective cohort study among Dutch workers on risk factors of musculoskeletal disorders. The study was approved by the medical ethical committee of the Netherlands organization for applied scientific research (TNO). The SMASH study, in which workers from 34 companies with both blue-collar and white-collar jobs from different parts of the Netherlands participated, has been described in more detail previously [15, 22].

At baseline 1990 of the 2,048 workers who were invited for the study participated. 1,802 (91 %) of these workers completed all questionnaires at baseline. Forty-six workers were excluded because they had been employed in their current job <1 year or had been working <20 h a week. Eleven workers were excluded because they had another paid job for a substantial amount of time at another company than at which they were recruited. As a result, 1,745 workers were eligible to participate in the current study. Descriptive statistics of these workers are provided in Table 1.

**Table 1:** Descriptive statistics (number of workers, gender, age, working hours per week and years of employment in the current job) of group of the workers who were eligible to participate in the current study (left column), workers of whom data were included in the statistical analysis (middle column) and workers of whom data were excluded from the statistical analysis (right column)

	Baseline workers	Workers in analysis	Workers not in analysis
N	1,745	1,086	659
Gender	m = 1,222 (71 %)/f = 510	m = 759 (70 %)/f = 327	m = 463 (72 %)/f = 183
Age (years)	35.9 ± 8.4	35.6 ± 8.7	35.4 ± 8.9
Hours per week	38.3 ± 4.5	38.2 ± 4.7	38.2 ± 4.7
Years in current job	9.9 ± 7.7	9.6 ± 7.6	9.5 ± 8.0

### Data Collection

At baseline, a number of potential risk factors were measured; questionnaire data were collected and assessment of physical load at the workplace was performed. Furthermore, a 3-year follow-up was conducted in which the prevalence of LBP was assessed annually.

Physical work load was assessed by video-observations and force measurements at the workplace. External force exertion at the hands was measured using force transducers or a weighting scale. Furthermore, workers were video-recorded at their workplace during 4 randomly selected moments of a workday. Each video-recording lasted 5–14 min, depending of the variability in working tasks. Thirty-five observers were recruited from a group of university students of the Faculty of Human Movement Sciences from the VU University Amsterdam. These observers had considerable knowledge on human kinematics and were trained using a standardized protocol to perform structured postural observations. These well-trained observers allocated all workers in task groups based on similar tasks and loads according to the International Standard Classification of Occupations. A continuous systematic observation of the video-recordings was used to assess trunk sagittal flexion, arm sagittal elevation, trunk rotation (in the transverse plane) and the presence of an external force in one-fourth of the workers of each task group. Furthermore, the time spend in a sitting position was observed. All data were extrapolated to an 8 h work day. A detailed description of these procedures was given by Hoogendoorn et al. [15].

Personal factors such as age and gender were assessed using self-administered questionnaires. A Dutch version of the Karasek’s Job Content Questionnaire for psychosocial work characteristics was used to assess job demands, decision authority, co-worker support and supervisor support [23]. The psychometric properties and the construction of these scales have been described by de Jonge et al. [24]. Exercise behaviour during leisure time was assessed with the Leisure Time Exercise Questionnaire [25]. Furthermore, driving a vehicle during work and during leisure time, flexion and rotation of the trunk and moving heavy loads during leisure time were assessed with the Loquest questionnaire [26]. A detailed description of all questionnaires has been given earlier [15, 22].

At baseline and at each year of the follow-up, the occurrence of LBP was assessed using a self-administered, adapted version of the Nordic Questionnaire [27]. LBP at baseline was defined when subjects reported regular or prolonged LBP in the previous 12 months before the start of the study. LBP during follow-up was defined as regular or prolonged LBP in the previous 12 months in at least one of the three annually follow-up questionnaires. The baseline population consisted of workers with and without LBP.

### Assessment of Low Back Load

For the assessment of CLBL during work, a manikin consisting of a trunk/head, upper arm and a lower arm/hand segment was constructed based on segment orientations obtained from the continuous video-observations (Table 2) and segment anthropometrics. As observed postures were supposed to be representative for the task group, average body weight and length within each task group were used for the estimation of segment anthropometrics (segment mass, length and centre of mass [28, 29]) and an estimation of the L5-S1 position [30] using regression equations.

**Table 2:** Observational categories

Variable	Observation		CLBL calculation
	Description	Category	Values
Trunk flexion (sagittal plane)	Neutral	<30°	0°
	Mild flexion	30°–60°	45°
	Extreme flexion	60°–90°	75°
	Very extreme flexion	>90°	90°
Trunk rotation (transverse plane)	Neutral	<30°	0°
	Twisting	>30°	30°
Arm elevation (sagittal plane)	Neutral	<30°	15°
	Mild elevation	30°–60°	45°
	Extreme elevation	60°–90°	75°
	Very extreme elevation	>90°	90°

The table shows a description and corresponding values for the observed variables. The last column shows body orientation values that were used for the calculation of CLBL

*CLBL* Cumulative low back load

For the complete observed period, a top–down calculation of net moments at the L5-S1 joint was performed using a general equation of motion [31]. In this calculation, segment gravitational forces of the constructed manikin combined with the measured external forces were taken into account. The calculated moments in the lower back were squared to accommodate for the fact that the moment levels have larger effect on injury risk than the number of repetitions [32]. Subsequently, CLBL was assessed by calculating the area under the moment curve. Mean task group values of the CLBL during the observed period were assigned to all workers in the same task group and were extrapolated to an entire work week based on the number of working hours of each individual in that task group during a week. All calculations were performed using custom developed Matlab software (version 7.7.0) [33].

### Statistical Analyses

The crude effect of CLBL (categorized into five categories, based on 20th percentiles –quintiles-) on LBP was assessed using a logistic regression with LBP during the follow-up (independent of LBP at baseline) as dependent variable, calculating ORs and corresponding 95 % CI. The choice for the number of categories is a balance between the power requirements (a sufficient number of workers in each category should remain) and optimizing contrast between the categories. The relationship of CLBL and LBP was checked on linearity by comparing regression coefficients between quintiles. In case of a linear relationship, logistic regression analyses were performed using CLBL as a continuous variable rather than categorised into five categories. In line with earlier reports on the present population [15], the variables age, gender, exercise behaviour during leisure time, quantitative job demands, decision authority, skill discretion, supervisor support, co-worker support, driving a vehicle during work and leisure time, flexion/rotation of the trunk during leisure time and moving heavy loads during leisure time were considered confounders. A second logistic regression analysis was performed to calculate ORs and corresponding 95 % CI for CLBL (independent variable) on LBP during the follow-up (dependent variable), adjusted for these confounders.

To compare the association of the dose measure CLBL with LBP during the follow-up to exposure measures reported earlier, six additional logistic regression analyses were performed. The earlier found risk factors percentage of the working time in a flexed position, number of lifts in an 8 h working day, and number of lifts ≥25 kg in an 8 h working day were used for comparison since they were reported to be significant risk factors for LBP in the same study population earlier [15]. In the first three analyses, the three exposures reported earlier were separately used as independent variables consecutively, without and with correction for CLBL. In the other three analyses CLBL was used as independent variable corrected for one of the three above mentioned physical risk factors, consecutively. Associations of all risk factors with LBP separately and corrected as indicated above were compared to assess the risk factor with the most consistent association with LBP. All statistical analyses were performed using SPSS (version 17.0.1).

## Results

### Population

Of the 1,745 workers eligible for participation in the current study, data on the physical load at workplace were available for 1,463 workers, while data on the occurrence of LBP in at least one follow-up measurement were available for 1,196 workers. For 1,192 workers, data on both physical load at workplace and on the occurrence of LBP were available. Of 1,086 workers, data on physical load at work, the occurrence of LBP and all confounders were available. 416 of these workers (38 %) reported LBP at baseline and 537 workers (49 %) reported LBP during at least one of the 3 years of follow-up. Data of these workers were used for further analysis (Table 1). In contrast to earlier work on the same population [15], workers with LBP at baseline were included in the present study.

### LBP Risk Model

The regression coefficients of the five CLBL categories, obtained from the logistic regression analyses, revealed a non-linear relationship of CLBL and LBP (Table 3). Therefore, categorised CLBL into quintiles (Table 4) was used as independent variable in the logistic regression models. A significant crude relation of CLBL and LBP in the group with the highest CLBL compared to the group with the lowest CLBL was shown (OR of 1.60, 95 % CI: 1.10–2.35). Also, CLBL adjusted for confounders yielded a significant relationship with the occurrence of LBP in the group with the highest CLBL compared to the group with the lowest CLBL (OR: 2.06, 95 % CI: 1.32–3.20; Table 3).

**Table 3:** Association of CLBL with LBP based on logistic regressions

Risk factor	LBP	No LBP	B	OR (95 % CI), n = 1,086^†^
*Regression model*				
CLBL				
1st quintile	109	107		Reference
2nd quintile	106	122	−0.15	0.86 (0.59–1.25)
3th quintile	93	129	−0.34	0.71 (0.49–1.04)
4th quintile	93	107	−0.15	0.86 (0.59–1.26)
5th quintile	136	84	0.47	1.60 (1.10–2.35)*
CLBL				
1st quintile				Reference^#^
2nd quintile			0.05	1.05 (0.70–1.58)
3th quintile			−0.13	0.87 (0.57–1.33)
4th quintile			0.03	1.03 (0.68–1.57)
5th quintile			0.72	2.06 (1.32–3.20)*

*B* regression coefficient, *OR* odds ratio, *CI* confidence interval

* Significant risk factor for LBP

^†^Of 1,086 workers data on the occurrence of LBP during follow-up, physical exposure at work and all confounders were available

^#^Logistic regression adjusted for the confounders: age, gender, exercise behaviour during leisure time, quantitative job demands, decision authority, skill discretion, supervisor support, co-worker support, driving a vehicle during work and leisure time, flexion/rotation of the trunk during leisure time and moving heavy loads during leisure time

*CLBL* Cumulative low back load

*LBP* Low back pain

**Table 4:** Category values of the five different categories (based on quintiles)

	n	Minimum	Maximum	Mean	SD
*Category values*					
1th quintiles	216	0.09	0.49	0.29	0.11
2nd quintiles	228	0.52	0.71	0.62	0.05
3th quintiles	222	0.74	1.13	1.03	0.13
4th quintiles	200	1.14	1.96	1.52	0.29
5th quintiles	220	1.99	10.83	3.65	2.38
Total	1,086	0.09	10.83	1.43	1.16

Number of subjects (n), minimum and maximum, mean and standard deviation of CLBL (all in MNm) in all five quintiles are listed

*CLBL* Cumulative low back load

*LBP* Low back pain

*SD* Standard deviation

To assess the predictive value of CLBL for LBP in comparison to exposures reported earlier, additional logistic regression analyses were performed in which these three risk factors were used as independent variables. Logistic regression analyses adjusted for confounders showed that all three risk factors significantly predicted LBP with ORs of 2.35 (1.46–3.79), 2.22 (1.33–3.36) and 2.38 (1.48–3.82) respectively in the most exposed groups (Table 5). However, when corrected for confounders and CLBL, only lifting >15 times ≥25 kg in an 8 h working day compared to no lifts of ≥25 kg was a significant risk factor for LBP (OR: 2.03 (1.23–3.36)), while percentage of the working time in a flexed position and number of lifts in a 8 h working day did not significantly predict LBP. Moreover, when separately corrected for each of these three risk factors, the CLBL remained a significant predictor for LBP in the group with the highest CLBL compared to the group with the lowest CLBL, showing ORs of 1.89 (1.04–3.45), 1.96 (1.15–3.36) and 1.85 (1.17–2.92) respectively (Table 5).

**Table 5:** Associations of the three earlier found risk factors (percentage of the working time in a flexed position, number of lifts in a 8 h working day, number of lifts ≥25 kg in a 8 h working day) with LBP based on logistic regression, adjusted for confounders (left columns) and adjusted for confounders and CLBL (right columns). Besides, association of CLBL with LBP adjusted for all earlier found risk factors separately are shown

Risk factor	LBP	No LBP	OR (95 % CI), n = 1,086^†^	OR (95 % CI), n = 1,086^†^
*Time in trunk flexion*				
≤5 % time ≥30°	256	287	Reference^a^	Reference^b^
5–10 % time ≥30°	96	110	1.01 (0.73–1.47)	1.15 (0.74–1.78)
>10 % time ≥30° & ≤5 % time ≥60°	120	120	1.15 (0.83–1.58)	0.91 (0.57–1.46)
>5 % time ≥60°	65	32	2.35 (1.46–3.79)*	1.45 (0.77–2.73)
*Number of lifts*				
Never	151	161	Reference^a^	Reference^b^
Never ≥10 kg/working day	81	94	0.74 (0.50–1.09)	0.69 (0.45–1.06)
Never ≥25 kg/working day	146	156	0.96 (0.68–1.36)	0.77 (0.51–1.17)
1–15 times ≥25 kg/working day	96	107	0.86 (0.59–1.27)	0.73 (0.44–1.19)
>15 times ≥25 kg/working day	63	31	2.22 (1.33–3.72)*	1.60 (0.88–2.92)
*Number of lifts* ≥*25* *kg*				
Never	378	411	Reference^a^	Reference^b^
1–15 time/working day	96	107	0.93 (0.67–1.29)	0.92 (0.63–1.34)
>15 times/working day	63	31	2.38 (1.48–3.82)*	2.03 (1.23–3.36)*
*CLBL*				
1st quintile				Reference^1^
2nd quintile				1.06 (0.70–1.59)
3th quintile				0.83 (0.51–1.33)
4th quintile				1.03 (0.60–1.78)
5th quintile				1.89 (1.04–3.45)*
*CLBL*				
1st quintile				Reference^2^
2nd quintile				0.97 (0.62–1.51)
3th quintile				0.88 (0.55–1.41)
4th quintile				1.05 (0.62–1.76)
5th quintile				1.96 (1.15–3.36)*
*CLBL*				
1st quintile				Reference^3^
2nd quintile				1.06 (0.71–1.60)
3th quintile				0.85 (0.56–1.31)
4th quintile				0.99 (0.62–1.57)
5th quintile				1.85 (1.17–2.92)*

*B* regression coefficient, *OR* odds ratio, *CI* confidence interval

* Significant risk factor for LBP

^†^Of 1,086 workers data on the occurrence of LBP during follow-up, physical exposure at work and all confounders were available

^a^Adjusted for the confounders: age, gender, exercise behaviour during leisure time, quantitative job demands, decision authority, skill discretion, supervisor support, co-worker support, driving a vehicle during work and leisure time, flexion/rotation of the trunk during leisure time and moving heavy loads during leisure time

^b^Adjusted for both above mentioned confounders and CLBL

^1^Adjusted for both above mentioned confounders and ‘Percentage of the working time in a flexed position’

^2^Adjusted for both above mentioned confounders and ‘Number of lifts in an 8 h working day’

^3^Adjusted for both above mentioned confounders and ‘Number of lifts ≥25 kg in an 8 h working day’

*CLBL* Cumulative low back load

*LBP* Low back pain

## Discussion

The first aim of the present study was to investigate whether a low back load dose, in this study expressed in CLBL is a predictor for LBP among workers. In the results, CLBL showed a significant association with the occurrence of LBP in the group with the largest CLBL. From these findings we can conclude that CLBL is a significant predictor of LBP. However, a significantly higher risk of LBP is only shown in the group with the highest levels of CLBL, which are levels of 2.00 MNm and more. As an example, for a moderate lifting task that would lead to a low back load of 200 Nm, this level of CLBL will be reached when 2.000.000/200^2^ = 50 of these lifts are performed during a work week. Ergonomic interventions should therefore be targeted mainly to workers who encounter these levels of CLBL which can emerge from combinations of awkward postures and/or high exposure tasks at work.

The second aim, to compare the association with LBP of CLBL to risk factors reported earlier, was attained using additional logistic regression analyses. These results show that CLBL remains a significant risk factor of LBP when corrected for the earlier found risk factors. Moreover, while the risk factors reported earlier are significant risk factors for LBP when corrected for confounders, only one risk factor remains significant when corrected for both confounders and CLBL. From these results we can conclude that CLBL has a more consistent association with LBP than the risk factors time in a flexed position and number of lifts in a working day. This finding supports our hypothesis that a low back load dose measure provides a stronger relationship with LBP than exposure measures of low back load since several exposures (e.g. lifting and bending) are incorporated in the dose. The fact that the risk factor number of lifts ≥25 kg in an 8 h working day had a comparable association with LBP may indicate that this exposure metric reflects incidental peak loads which may constitute an independent risk for LBP. Again, this underscores the importance of focussing on peak loads.

### Methodological Considerations

The strength of the present study is that the results are based on a large prospective cohort study. This design, in which the prevalence of LBP was measured during a 3-year follow-up allows insight into potential causes of LBP [34]. Of the 1,745 workers who were eligible to participate in this study, data on physical load at the workplace, on the occurrence of LBP and on confounders were available for 1,086 workers. Selection or attribution bias may be possible due to this substantial loss to follow-up. However, the group of workers analyzed and the group of workers who were excluded from the statistical analysis due to incomplete data show comparable descriptive characteristics with respect to age, gender, working hours per weeks and years of employment (Table 1), thereby reducing the likeliness of these kinds of biases.

In contrast to earlier studies on this study population [15], workers suffering from LBP at baseline were included in our analyses. It has been shown that a history of LBP is a good predictor of future LBP since LBP often comes in several episodes [35, 36]. Excluding workers with pain at baseline thus seems unreasonable since it cannot be excluded that workers without complaints at baseline, have not had any complaints 2 or 3 years before the baseline measurements. Therefore, we can assume that when excluding these workers, the healthy worker effect will be reinforced. Besides, including workers with a history of LBP makes the present results applicable to a larger part of the working population since excluding these workers would reduces the external validity of the current results. Including workers with pain at baseline seems therefore reasonable. Furthermore, an extra analysis in which only the workers without baseline complaints were analyzed (i.e. the workers who did not report LBP at baseline) showed changes in ORs <0.1 in the associations of CLBL with LBP. These findings, showing that associations of CLBL and LBP do not change considerably, support the consistency of the current results.

A limitation of the present study is the subjective assessment of LBP. It has been shown that diagnosing LBP is complicated. However, subjectively assessed LBP has been shown to have a strong relation with clinically examined LBP [37] and sickness absence due to LBP [38]. Furthermore, the CLBL assessment method contains some limitations. First, observations based on videos may suffer from errors and potential bias [39]. Furthermore, movements which are not in the sagittal plane are difficult to assess [40] and the outcome of the measurement is dependent on the selected time at the measurement-day, the number of subjects per task group and the number of measurements per subject [41]. The latter problems were addressed by measuring workers at four random chosen moments of the day and measuring several workers in each task group, to obtain more precise estimates of the exposure within groups [42]. Structured postural observations have been performed by multiple observers. Although, it has been shown that postural video observations are reliable among observers in work-site situations [43, 44], inter-observer reliability was not evaluated in the group of observers we recruited. Therefore, because several trained observers classified the body postures, inter- and intra-observer variation cannot be ruled out.

Another source of error in our study might have emerged from the fact that workers were observed at four randomly chosen occasions of the work day for a finite amount of time rather than a complete observation of the whole work day. This choice was made based on a pilot study, in which it has been shown that the largest amount of variation in physical work exposure, is variation in exposure within workers rather than variation in exposure between workers [45]. The appropriateness of our measurement strategy was furthermore supported by showing small within group variability and large between group variability in data on the same cohort [22]. Measuring on multiple occasions on a single work day is therefore considered a feasible and justifiable approach to reduce the amount of observation time. Furthermore, it has been shown that measuring work load at four occasions during a day is sufficient to obtain a reliable estimate of the work exposure [41].

A final source of error of the CLBL assessment results from the biomechanical calculation, which contains assumptions concerning the workers’ anthropometrics and segment orientations. Furthermore, segment dynamics were not taken into account in this calculation, which may have led to an underestimation of the calculated low back load. The above mentioned sources of errors in the calculation of CLBL suggest that associations of dose measures with LBP might become even higher when more reliable dose estimates are available. Besides, as an indicator of back load, low back moments were used, although it may be argued that injury risk and thus potentially LBP is more accurately predicted by spinal forces, either in compression [46] or shear direction [18, 19]. However, a strong correlation of low back moments with shear forces and compression forces has been reported [47] reducing the risk of large errors due to the use of moments instead of spine forces.

### Comparison with Previous Findings

The relationship between awkward body postures during work (e.g. trunk flexion, trunk rotation and lifting) and LBP has been reported in several prospective studies in the last decades [13, 15]. However, several reviews [9, 11, 12, 48] showed that results are inconsistent. The association of low back load dose measures and the risk of LBP can give more insight in the aetiology of LBP. An association of cumulative and peak low back load with LBP has been has been described before [19–21]. However, these associations are based on retrospective studies. The present results are comparable to the earlier findings and thus confirm these findings in a prospective study, thereby providing strong support for a causal relationship between CLBL and LBP.

## Conclusions

From the current study it can be concluded that CLBL is a significant risk factor for LBP with more consistent associations with LBP than risk factors reported earlier. Moreover, CLBL appeared to reflect both the effects of working in a trunk flexed position and number of lifts during work on LBP risk. The risk factor number of lifts ≥25 kg had additional value in predicting the risk of LBP besides CLBL. The results of the present study may have implications for prevention programs for LBP. Interventions aimed at changes in posture and lifting forces, but also reduction of duration of exposure to adverse postures should, according to these findings be considered.
